# Isoniazid preventive therapy: Uptake, incidence of tuberculosis and survival among people living with HIV in Bulawayo, Zimbabwe

**DOI:** 10.1371/journal.pone.0223076

**Published:** 2019-10-03

**Authors:** Saziso Nyathi, Riitta A. Dlodlo, Srinath Satyanarayana, Kudakwashe C. Takarinda, Hannock Tweya, Sithokozile Hove, Ronnie Matambo, Winnie Mandewo, Khulamuzi Nyathi, Edwin Sibanda, Anthony D. Harries

**Affiliations:** 1 Health Services Department, City of Bulawayo, Bulawayo, Zimbabwe; 2 International Union Against Tuberculosis and Lung Disease (The Union), Paris, France; 3 International Union Against Tuberculosis and Lung Disease (The Union), Harare, Zimbabwe; 4 AIDS and TB Directorate, Ministry of Health and Child Care, Harare, Zimbabwe; 5 Light House Trust, Lilongwe, Malawi; 6 Department of Clinical Research, London School of Hygiene and Tropical Medicine, London, England, United Kingdom; 7 Elizabeth Glaser Peadiatric AIDS Foundation, Harare, Zimbabwe; University of Ghana College of Health Sciences, GHANA

## Abstract

**Setting:**

Four primary health care clinics providing tuberculosis (TB) and Human Immunodeficiency Virus care services in Bulawayo, Zimbabwe.

**Objectives:**

To assess isoniazid preventive therapy (IPT) initiation and completion, factors associated with IPT uptake and incidence of TB, and TB and antiretroviral treatment (ART) outcomes among people living with HIV (PLHIV).

**Design:**

This was a cohort study using routine data in the records for PLHIV initiated on ART from October 2013 to March 2014 with 31 December 2017 as the end of the follow-up period.

**Results:**

A total of 408 PLHIV were eligible for IPT, 214 (52%) were initiated on IPT and 201 (94%) completed IPT. No person in the IPT-initiated group developed Tuberculosis (TB). Six persons with TB were reported among the non-IPT-initiated group leading to an incidence of 9 cases/1,000 person-years of follow-up. About 70% of those who developed and were treated for TB had a successful TB treatment outcome. The survival on ART at four years of follow-up was 88% among the IPT-initiated PLHIV that was significantly higher than the 75% survival in the group not- initiated on IPT.

**Conclusion:**

The study revealed low IPT initiation among eligible PLHIV who, if started on IPT, completed the six month regimen. TB was reported only among the PLHIV not-initiated on IPT and the four year ART survival was higher in the IPT-initiated group than in the non-initiated group. These findings reinforce the need to strengthen IPT uptake among PLHIV in Bulawayo.

## Introduction

Globally, in 2017, there were an estimated 37 million people living with HIV (PLHIV). Of these, 22 million were on antiretroviral therapy (ART) [1]. In the same year, globally, there were an estimated 10 million incident cases of tuberculosis (TB). In PLHIV, TB is one of the major causes of morbidity and mortality accounting for approximately one in three AIDS-related deaths [1]. PLHIV with TB infection have a 10% annual risk of developing TB compared to 10–20% lifetime risk in HIV non-infected individuals [2,3]. An estimated 1.4 million PLHIV developed TB and 0.38 million PLHIV died from TB in 2016 [1,3,4]. By providing Isoniazid Preventive Therapy (IPT) to PLHIV, the occurrence of TB can be reduced by up to 60% and TB-related death rates by approximately 40% [5].

ART has a potent TB preventive effect in PLHIV and reduces the individual risk of TB [6]. IPT, however, augments this protection by a further 35% [7,8]. An observational study and a randomised controlled trial in Africa have both shown that IPT results in a significant reduction in death independent of ART [9,10]. This compelling evidence strongly supports the provision of IPT as an integral part of the international guidelines for TB-HIV collaborative activities [11].

IPT roll-out has been slow in several high HIV and TB burden countries with the IPT coverage among PLHIV ranging from 2.4% to 73% in 2016 [4,12]. This has been attributed to several operational challenges in implementing IPT services especially in resource limited settings. These challenges include difficulties in diagnosing TB infection, difficulties in ruling out active TB disease, uncertainty about the optimal time to initiate IPT, concerns about the development of isoniazid mono-resistance and isoniazid induced liver toxicity and unclear guidelines about the optimal duration of IPT, i.e., whether IPT should be given for 6 months, 9 months, 36 months or lifelong [11]. Some researchers infer that 6 months of IPT might be insufficient in countries with a high burden of TB such as in Southern Africa [5]. Apart from these issues, drug shortages, poor IPT adherence and completion rates have also been reported by several studies [13].

Given this scenario, there is a relative dearth of information on the effectiveness of IPT in preventing TB disease under routine programmatic conditions in various settings. The current recommendation for all countries implementing IPT services is to monitor the IPT uptake and completion rates, and, assess the effectiveness of IPT in preventing TB disease and improving survival under routine programmatic conditions. This context specific information will help countries make better decisions about the optimal stratagies for implementing and/or scaling up IPT services in their settings [5].

Zimbabwe, a country in southern Africa, with an estimated population of 13.6 million in 2017 [14], has experienced a severe HIV-associated TB epidemic. IPT services for PLHIV were rolled out in the country in 2012 with the drug being given for a period of 6 months. A previous study reported that ~80% of PLHIV initiated on IPT completed the six-months [15]. To our knowledge, however, there are no studies from Zimbabwe that have assessed whether all eligible PLHIV are initiated on IPT and the incidence of TB among those initiated on IPT under routine programmatic settings. In order to fill this information gap, we conducted an operational research study in Bulawayo, with two aims: a) to assess the IPT initiation and completion rates among PLHIV eligible for IPT and the patients’ characteristics associated with IPT uptake; and b) to assess the incidence of TB, the TB treatment outcomes and ART outcomes among those initiated and not initiated on IPT over a 4 year follow-up time period.

## Methods

### Design

This was a retrospective cohort study involving secondary analysis of routinely collected data.

### Study setting

In Zimbabwe, in 2017 the general population prevalence of HIV was 13.9%, the incidence of TB was 212 per 100,000 population and 1.8–4.6% of TB patients had multidrug-resistant TB (MDRTB) [16]. In 2011, IPT was incorporated into the national TB-HIV guidelines. IPT services were rolled out in 2012 intially to 10 public health sector clinics and later expanded to all health facilities in the country. According to these guidelines, all PLHIV without symptoms of TB or current TB disease, without active liver disease, who have had ART for a minimum of three months and no more than three years were eligible for IPT. The IPT regimen consisted of isoniazid 5mg/kg body weight up to 300mg per day, self administered, for 6 months supplemented with pyridoxine 25–50 mg/day [15]. All PLHIV initiated on IPT received adherence counselling prior to initiation of IPT and this was repeated every month when they came to collect their isoniazid supplies. During the course of IPT, they were also monitored for the occurance of TB disease and/or liver toxicity and IPT was stopped in the presence of any of these two events [17,18]. After the completion of IPT all PLHIV were monitored for the occurance of TB disease during the quarterly ART follow-up visits using a structured TB symptom screening tool. Those with TB symptoms were investigated / examined for TB either by sputum examination through Xpert MTB /RIF or radiological examination. All information about IPT eligibility, IPT initiation, adherence, IPT outcomes and the occurance of TB disease were recorded in the opportunistic infection (OI)/ART patient booklets that were maintained at the health facility for each PLHIV.

### Study site

The study was conducted in Bulawayo which is the second largest city in Zimbabwe with an estimated population of 686,000 [14]. By the end of 2017, approximately 80,000 PLHIV were in HIV care in different health facilities in the city. The Health Services Department (HSD) of the City of Bulawayo provides primary health care services through a network of 19 clinics and one infectious diseases hospital [12]. According to the national guidelines, IPT services were initiated as a pilot project in three clinics (Luveve, Magwegwe and Emakhandeni) in December 2012 and by June 2017, all clinics were providing these services. By December 2018 there were approximately 22,000 PLHIV in Bulawayo who had been initiated on IPT [12].

### Study participants and study period

In order to simultaneously accommodate the assessement of IPT uptake and the occurence of TB over a 3–4 year follow-up period within the same cohort, we included all PLHIV (aged 18 years and above) initiated on ART from 1 October 2013 to 31 March 2014 in four clinics in Bulawayo namely, Emakhandeni, Entumbane, Luveve and Nketa Clinics. In these facilities, we reviewed OI/ART patient booklets of all those who were initiated on ART during the above mentioned time period. We took a cut-off date of 31 December 2017 as the end of the follow-up period to assess the incidence of TB and ART outcomes in our study participants.

### Data variables, collection and data sources

We collected individual patient-specific information on demographic and clinical characteristics, IPT eligibility, IPT initiation, IPT completion, the occurrence of TB, TB treatment outcomes (in those with TB disease) and ART outcomes at the end of the follow-up period. ART outcomes were assessed at the end of the follow up period. The outcomes that were used in the study include: a) alive on ART, if the patient was alive and continuing on ART at the end of the follow up period; b) died; c) lost to follow up; and d) transferred out. The primary source of data was OI/ART patient booklets. In patients who developed TB disease, we additionally reviewed the presumptive TB register to ascertain if those that were presumed to be having TB were investigated for TB and rifampicin. The TB health facility register was checked for TB treatment initiation, completion and the TB treatment outcomes. The data were collected from November to December 2018, extracted from the sources and then double-entered into a structured proforma using EpiData Entry software (Version 2.2.2.182, EpiData Association, Odense, Denmark) and validated.

### Analysis and statistics

We summarised the demographc and clinical characteristics of the study population using numbers and proportions. We assessed the association between patient characteristics with the IPT initiation using relative risks and adjusted relative risks. Bivariable and multivariable binomial log models were used to calculate these relative risks. We assessed the TB incidence rate in PLHIV who were initiated and not initiated on IPT as the number of cases per 1000 person years of follow-up. For estimating the person years of follow-up (the denominators for the incidence rate), we used the date of IPT eligibility as the ‘start date’ and the date of TB diagnosis or the date of ART outcome (death/loss to follow-up or transferred out) or the censoring date (31 December 2017)—whichever was earlier—as the ‘end-date’. Levels of significance were set at 5% (<0.05) for statistical significance. Data were analysed using two statistical softwares, namely EpiData Analysis (Version 2.2.2.182, EpiData Association, Odense, Denmark) and Stata (Version 15, StataCorp, College Station, Texas, USA).

### Ethics approval

We obtained ethics approval to conduct the study from the Ministry of Health and Child Care (MOHCC), Zimbabwe, Medical Research Council of Zimbabwe (approval number E221). Ethics approval was also obtained from the Ethics Advisory Group, of The Union, Paris, France (EAG Number 54/18). Since our study involved review of patient records without direct patient contact, we received a waiver from obtaining informed consent from the study participants from the MRCZ and EAG.

## Results

**Fig 1:**
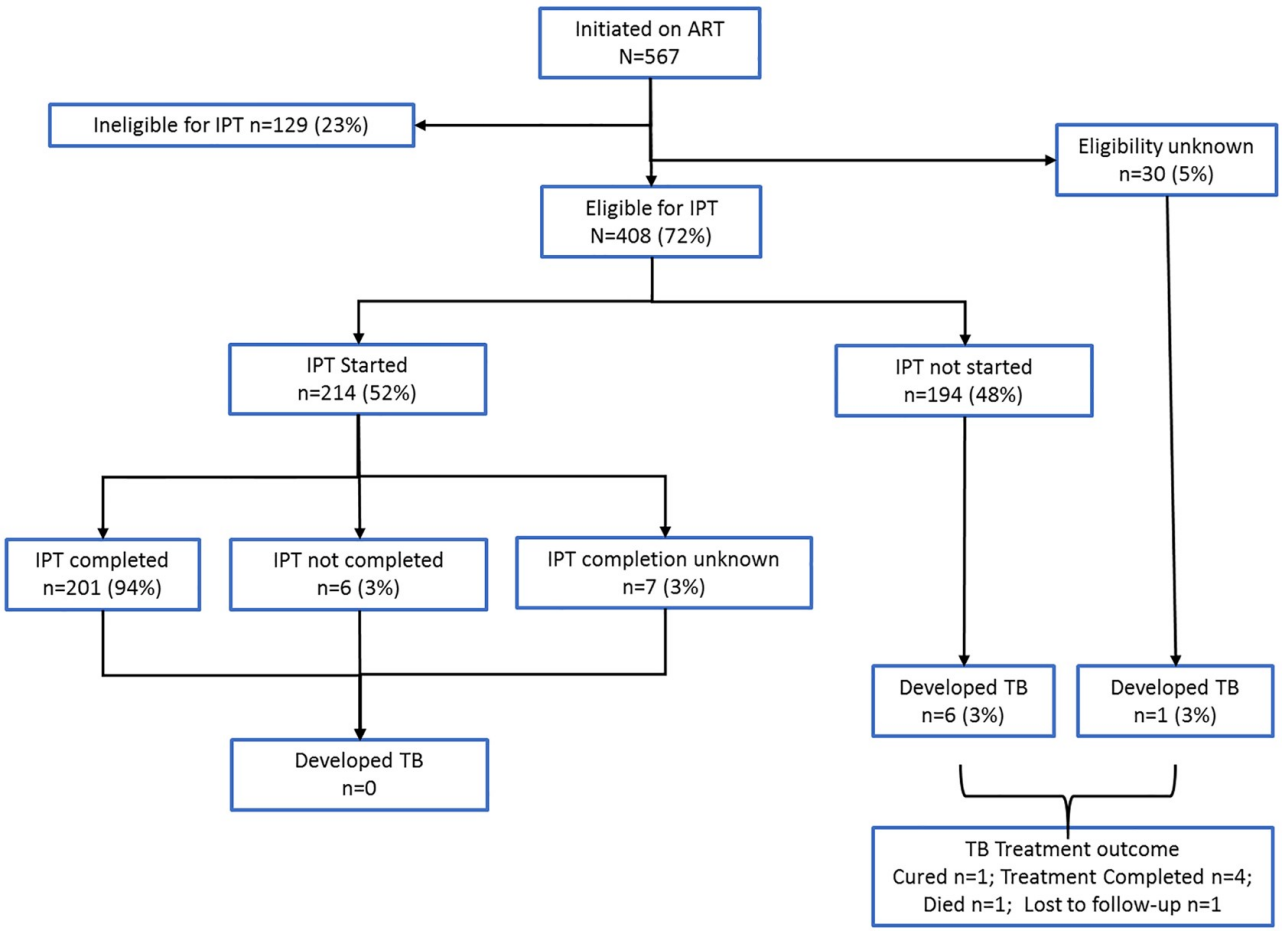
Flow diagram of persons living with HIV who were assessed and initiated on Isoniazid Preventive Therapy in Bulawayo, Zimbabwe from October 2013 to March 2014. IPT = Isoniazid Preventive Therapy; PLHIV = People Living with HIV; ART = Antiretroviral Therapy; TB = Tuberculosis.

Of 567 PLHIV initiated on ART, 129 were ineligible for IPT (due to the presence of TB disease) and the IPT eligibility and initiation status was not recorded for 30 PLHIV. A total of 408 PLHIV were therefore recorded as eligible for IPT and 214 (52%) were initiated on IPT (Fig 1). Of those initiated on IPT, 201 (94%) completed IPT.

The characteristics of IPT eligible PLHIV (N = 408) are described in Table 1. About 76% were in the age group 20–60 years, 62% were females, 65% were married, 66% had CD4 cell counts between 100–350 cells/mm^3^ and 34% were in WHO clinical stage 3 and 4.

**Table 1:** Characteristics of PLHIV who were initiated on ART in Bulawayo, Zimbabwe from October 2013 to March 2014 and eligible for IPT.

Characteristics	Total	
	N	(%)
**Total**	**408**	(100)
**Age group (in Years)**		
<20	9	(2.2)
20–24	20	(4.9)
>24–49	311	(76.2)
> = 50	65	(15.9)
Not recorded	3	(0.7)
**Gender**		
Male	148	(36.3)
Female	256	(62.7)
Not recorded	4	(1.0)
**Marital Status**		
Married	266	(65.2)
Single	86	(21.1)
Widowed	34	(8.3)
Separated/divorced	19	(4.7)
Not recorded	3	(0.7)
**CD4 Cell count (cells/mm3)**		
> = 350	16	(3.9)
200 to <350	169	(41.4)
100 to <200	101	(24.8)
<100	92	(22.5)
Not recorded	30	(7.4)
**WHO Staging**		
Stage 1	123	(30.1)
Stage 2	145	(35.5)
Stage 3	117	(28.7)
Stage 4	23	(5.6)

IPT = Isoniazid Preventive Therapy; PLHIV = People Living with HIV; ART = Antiretroviral Therapy

The characteristics associated with IPT initiation are given in Table 2. Those who were separated/divorced were 58% more likely to initiate IPT compared to those who were married [adj RR 1.58; 95% CI: 1.20–2.01]. Apart from this characteristic, no other characteristics were statistically associated with IPT initiation.

**Table 2:** Characteristics associated with IPT initiation in PLHIV who were initiated on ART in Bulawayo, Zimbabwe from October 2013 to March 2014.

Characteristics	Total	Initiated on IPT		RR (95% CI)	Adj RR (95% CI)	P-Value
	N	n	(row %)			
**Total**	**408**	214	(52.5)			
**Age group (in Years)**						
<20	9	4	(44.4)	0.85 (0.40–1.78)	0.72 (0.33–1.59)	0.424
20–24	20	12	(60.0)	1.15 (0.79–1.67)	1.12 (0.77–1.64)	0.526
>24–49	311	162	(52.1)	Reference	Reference	
> = 50	65	34	(52.3)	1.00 (0.77–1.29)	1.07 (0.81–1.40)	0.611
Not recorded	3	2	(66.7)	0.52 (0.46–0.59)	1.52 (0.77–2.99)	0.218
**Gender**						
Male	148	70	(47.3)	0.85 (0.70–1.05)	0.86 (0.70–1.07)	0.188
Female	256	141	(55.1)	Reference	Reference	
Not recorded	4	3	(75.0)	1.36 (0.76–2.42)	1.37 (0.72–2.58)	0.325
**Marital Status**						
Married	266	132	(49.6)	Reference	Reference	
Single	86	50	(58.1)	1.17 (0.95–1.45)	1.17 (0.93–1.47)	0.161
Widowed	34	15	(44.1)	0.89 (0.59–1.32)	0.83 (0.54–1.27)	0.404
Separated/divorced	19	15	(78.9)	1.59 (1.22–2.06)	1.62 (1.24–2.12)	**<0.001**
Not recorded	3	2	(66.7)	1.34 (0.59–3.02)	1.46 (0.69–3.10)	0.317
**CD4 Cell count (cells/mm3)**						
> = 350	16	7	(43.8)	Reference	Reference	
200 to <350	169	102	(60.4)	1.37 (0.78–2.43)	1.28 (0.73–2.26)	0.383
100 to <200	101	47	(46.5)	1.06 (0.58–1.92)	0.96 (0.53–1.74)	0.906
<100	92	41	(44.6)	1.01 (0.55–1.85)	0.96 (0.52–1.76)	0.911
Not recorded	30	17	(56.7)	1.29 (0.68–2.45)	1.24 (0.64–2.35)	0.528
**WHO Stage**						
Stage 1	123	66	(53.7)	Reference	Reference	
Stage 2	145	80	(55.2)	1.02 (0.82–1.28)	1.09 (0.87–1.36)	0.433
Stage 3	117	57	(48.7)	0.91 (0.70–1.16)	0.96 (0.78–1.25)	0.798
Stage 4	23	11	(47.8)	0.89 (0.56–1.40)	0.95 (0.59–1.55)	0.862

IPT = Isoniazid Preventive Therapy; PLHIV = People Living with HIV; ART = Antiretroviral Therapy; RR = Relative Risk; 95% CI = 95% Confidence Intervals

Seven PLHIV developed TB (six from among the group of IPT eligible PLHIV who were not initiated on IPT and one from the IPT eligibility unknown group). Of these seven, six were new pulmonary TB patients (one with bacteriologically confirmed drug-susceptible TB and five with clinically diagnosed TB) and one was a new extra-pulmonary TB patient. All patients were treated with the first line anti-TB treatment regimen. Five had a successful TB treatment outcome (cured or treatment completed) and two had an unfavorable outcome (died, lost to follow-up) (Fig 1).

The 408 IPT eligible PLHIV contributed 1,465 person-years of follow-up up to the censor date—815 and 650 person years of follow-up in the IPT initiated and non-initiated groups, respectively. The TB incidence in the IPT initiated group was zero and in the IPT non-initiated group it was nine [95% CI: 4–20] per 1,000 person years of follow-up.

There were 19 PLHIV in this IPT eligible cohort who were transferred out before the censor date (31 December, 2017). Among the remaining 389 PLHIV, the ART outcomes at the end of the follow-up period were: alive and on ART = 319 (82%), lost-to-follow up = 56 (14%) and died = 14 (4%). The proportion alive and on ART in the IPT initiated and not initiated groups were 88% and 75%, respectively. After adjusting for age group, sex, marital status, CD4 cell counts and WHO clinical stage, the proportion alive and on ART in the IPT initiated group was statistically higher than in the IPT not initiated group [adj RR: 1.15; 95% CI: 1.04–1.27] (Table 3).

**Table 3:** Characteristics associated with retention in care by the end of 2017 among IPT eligible PLHIV who were initiated on ART in Bulawayo, Zimbabwe from October 2013 to March 2014.

Characteristics	Total	Alive and on ART		RR (95% CI)	Adjusted RR (95% CI)	P-value
	N	n	(Row%)			
**Total**	389*	319	(82.0)			
**IPT Initiation**						
Yes	207	182	(87.9)	**1.16 (1.06–1.28)**	**1.15 (1.04–1.27)**	**0.003**
No	182	137	(75.3)		**Reference**	
**Age group (in Years)**						
<20	9	7	(77.8)	0.95 (0..67–1.36)	0.93 (0.64–1.34)	0.701
20–24	19	16	(84.2)	1.03 (0.84–1.26)	1.02 (0.84–1.23)	0.831
>24–49	295	240	(81.4)	Reference	Reference	
> = 50	64	54	(84.4)	1.03 (0.92–1.16)	1.06 (0.93–1.20)	0.355
Not recorded	2	2	(100.0)	1.22 (1.16–1.29)	1.30 (1.13–1.49)	<0.001
**Gender**						
Male	140	110	(78.6)	0.94 (0.84–1.00)	0.95 (0.85–1.06)	0.366
Female	245	205	(83.7)	Reference	Reference	
Not recorded	4	4	(100.0)	1.19 (1.13–1.26)	**1.17 (1.05–1.29)**	**0.003**
**Marital Status**						
Married	255	205	(80.4)	Reference	Reference	
Single	82	68	(82.9)	1.03 (0.91–1.15)	1.02 (0.90–1.15)	0.728
Widowed	33	29	(87.9)	1.09 (0.95–1.25)	1.05 (0.91–1.22)	0.432
Separated/divorced	16	14	(87.5)	1.08 (0.89–1.32)	1.05 (0.87–1.26)	0.58
Not recorded	3	3	(100.0)	1.24 (1.17–1.32)	1.27 (1.09–1.47)	0.001
**CD4 Cell count (cells/mm3)**						
> = 350	16	13	(81.3)	Reference	Reference	
200 to <350	161	133	(82.6)	1.01(0.79–1.30)	0.96 (0.75–1.23)	0.797
100 to <200	99	79	(79.8)	0.98 (0.76–1.26)	0.95 (0.73–1.23)	**0.72**
<100	84	71	(84.5)	1.04 (0.80–1.33)	1.03 (0.80–1.33)	0.781
Not recorded	29	23	(79.3)	0.97 (0.72–1.31)	0.95 (0.71–1.28)	0.759
**WHO Staging**						
Stage 1	119	98	(82.4)	Reference	Reference	
Stage 2	141	121	(85.8)	1.04 (0.93–1.15)	1.03 (0.93–1.15)	0.506
Stage 3	106	82	(77.4)	0.93 (0.82–1.07)	0.92 (0.80–1.06)	0.293
Stage 4	23	18	(78.3)	0.95 (0.75–1.19)	0.93 (0.74–1.18)	0.593

*excluding 19 patients who were transferred out.

PLHIV = People living with HIV; ART = Antiretroviral therapy; IPT = Isoniazid Preventive therapy; RR = Relative risk; aRR = Adjusted Relative risk.

## Discussion

This is the first study from Zimbabwe that has assessed IPT eligibility, IPT initation and completion rates, TB incidence, TB treatment outcomes and ART outcomes under routine programme conditions all within the same cohort of PLHIV over a four year period.

The study shows that nearly three out of four patients initiated on ART between October 2013 to March 2014 were eligible for IPT according to the national guidelines in place at that time. Of those who were eligible, only half were initiated on IPT with 94% IPT completion rates. No person in the IPT initiated group developed TB but in the IPT non-initiated group six persons reportedly developed TB. About 70% of those who developed TB had a successful TB treatment outcome. The overall survival on ART for this cohort of IPT eligible patients at the end of four years was 82% with the survival higher in the group that was initiated on IPT.

The major strengths of this study was that it was conducted using routine programme records and therefore the results are likely to reflect ground level realities. The study also adhered to the Strengthening on the Reporting of OBservational Studies in Epidemiology (STROBE) guidelines[19,20]

There were four major limitations of the study. First, the routine programme records may have contained ‘data recording errors’. We tried to minimise these errors by selecting relatively better performing clinics of Bulawayo with good record keeping practices. We also adopted the practice of double data entry and validation to minimize the data entry errors. However, we did not contact a sub-sample of the study participants to verify whether their details in the records were correct or not. We are therefore unable to rule out or quantify the magnitude of “data recording errors’. Second, we had missing data. We could not ascertain IPT eligibility, IPT initiation or completion status from the records for 30 study participants. Instead of excluding these individuals, we examined their records and one of them had developed TB. We included this information in our results in Fig 1. These persons and others could have accessed IPT services in other health facilities without informing providers at the clinics where they were started on ART. Similarly, there were a few study participants who had missing data on key study variables. We could not make the assumption that missing data were completely at random. Therefore, instead of excluding these patients we created an additional category within each variable for missing data and performed the analysis. This may not have completely removed the biases in our study results due to missing data. Third, our study variables were restricted to routinely collected data within the programme. Several other variables, such as socio-economic status of patients, previous history of TB, health facility level factors (such as training status of staff, availability of adequate drug supplies) are also known to influence the uptake of IPT services. As we did not capture this information, we were unable to account for these factors in our analysis or interpretation of the study results. There could be residueal confounding as a result of these unmeasured variables. Fourth, since we selected relatively better performing health clinics of Bulawayo, the study findings may not be generalisable to other health facilities in the country.

Despite these limitations, the study results have the following five implications for policy and practice.

First, nearly three out of four PLHIV enrolled for ART during the selected time period were eligible for IPT. Other studies from Africa show that the proportion of PLHIV eligible for IPT ranges from 78% to 90% [21,22]. Since 2014, the criteria for enrolment into ART has changed and currently the country follows the ‘test and treat approach’for ART [23] wherein all PLHIV are eligible for ART irrespective of their clinical status or CD4 Cell count levels [23]. Therefore the proportion of IPT eligible under the current programmatic conditions could be higher than what we found in our study. It is also noteworthy that ~20% of PLHIV initiating ART in this study were not eligible for IPT due to active TB. This also suggests missed opportunity for earlier initiation of ART and IPT.

Second, only about half of the IPT eligible PLHIV were initiated on IPT. This is within the range that has been found in other studies in Africa from 2014–2017 [15,24,25]. Our study results also showed that PLHIV who were separated or divorced were more likely to be initiated on IPT. The reasons for higher initiation of IPT in separated or divorced inviduals is not fully known and this could very well be “by-chance” and not a deliberate human effort. Anecdotal evidence indicates that ART providers were cautious in initiating IPT in clinically unwell PLHIV especially in those with low CD4 cell counts due to possible undiagnosed TB. In our study we did not find an association between CD4 Cell count and IPT initiation. Studies from other parts of Africa also show that isoniazid drug shortages, fear of creating isoniazid resistance, problems in patient acceptance, and lack of commitment of health care workers are factors responsible for not initiating eligible PLHIV on IPT [25–27]. Similar issues/concerns may also be prevalent in our setting.

Third, the IPT completion rates were high with 94% of those initating on IPT completing the six-month treatment. This is much higher than what has been reported previously elsewhere in Africa and Zimbabwe where completion rates have ranged from 75–85% [15,22,28]. The reasons for high completion rates in our settings can be attributed to the pre-treatment and periodic on-treatment counselling services, and the integration of tuberculosis and HIV services [29]. Prior studies in Malawi, Tanzania, South Africa and Botswana indicate that if patients are initiated on IPT whilst having good knowledge of the benefits of ART and IPT they are likely to be more adherent [25–27].

Fourth, there was a large difference in TB incidence between those initiated on IPT and those not initiated on IPT, with no cases of TB diagnosed in the former group. Because of the small sample size we were unable to quantify the exact magnitude of the risk rate difference (and its 95% CI) between both the groups. Nevertheless, we feel that most of these TB cases in the non-IPT group can be attributed to non-initiation of IPT since both the groups were on ART and if we presume that there was no residual confounding between those initated on non-initiated on IPT. These cases represent missed oppurtunities for TB prevention. About 70% of those who developed TB had successful TB treatment outcomes which was less than what has been reported about TB treatment outcomes in PLHIV on ART in Zimbabwe and Africa which ranges from 78% to 83% [3,30,31].

Finally, our study results also show that the 4 year survival of PLHIV on ART and IPT was better than those who were not initiated on IPT. This is in line with other studies from Africa that have shown that provision of IPT along with ART not only prevents TB, but also has other survival benefits depending upon the community settings in which IPT services are implemented [9, 10, 19, 30, 32]. We could not investigate whether this is due to increased protection against TB disease or due to some other pathway in our study due to the small sample size (zero events in the IPT initiated group). This is an important subject for future research.

**In conclusion**, this study showed that about half of PLHIV eligible for IPT were initiated on IPT. The IPT completion rates were much higher than what has been reported in other studies. TB was observed only in those not initiated on IPT and 4 year ART survival was higher in IPT initiated group compared with the non-initiated group. These study findings reinforce the need to improve the IPT coverage among PLHIV in the country.
